# Interventions to improve teamwork and wellbeing in primary care settings: a mixed method review

**DOI:** 10.1093/eurpub/ckac129.043

**Published:** 2022-10-25

**Authors:** M Panagioti, N Tyler, A Hodkinson

**Affiliations:** Manchester University, Manchester, UK

## Abstract

**Background:**

General practices are experiencing increasing pressures due to rising demand, declining staff numbers, and knock-on impacts on patient care. The COVID-19 pandemic has added further challenges and reinforced the importance of teamwork and organisational settings. We undertook a mixed-method systematic review to explore which interventions can improve teamwork within primary care and improve inter-sector partnerships with other health and social care services.

**Methods:**

Five major bibliographic databases were systematically searched for relevant studies from inception to February 2022. We included controlled intervention study designs and linked qualitative studies. For amenable data, meta-analysis is being undertaken using random effects models taking into account the between study heterogeneity (quantified using the I2 statistic) and potential publication bias (funnel plots and Egger's test). The qualitative studies are analysed using thematic analyses.

**Results:**

The original search yield of 3012 studies, of which 14 studies with 1,534 participants were include in our analyses. Most of the evaluated interventions focused on improving non-technical skills and provided evidence of improvements in the quality of teamwork in primary care. Meta-analysis and narrative synthesis is undertaken to examine the impact of the teamwork interventions on staff outcomes (team attitudes, knowledge, and functioning; wellbeing), and patient outcomes (e.g. quality of patient care, patient satisfaction/experience).

**Conclusions:**

The findings provide information of immediate importance for the mental health and wellbeing and teamwork support of professionals entering primary care and for the organisation of primary care services.

